# Utility of Alternative Effect Size Statistics and the Development of a Web-Based Calculator: *Shiny-AESC*

**DOI:** 10.3389/fpsyg.2018.01221

**Published:** 2018-07-17

**Authors:** Don C. Zhang

**Affiliations:** Psychology, Louisiana State University, Baton Rouge, LA, United States

**Keywords:** effect size statistics, validity, visual-aids, decision-aids, shiny *R*, science communication

## Abstract

Alternative displays of effect size statistics can enhance the understandability and impact of validity evidence in a variety of applied settings. Arguably, the proliferation of alternative effect size statistics has been limited due to the lack of user-friendly tools to create them. Common statistical packages do not readily produce these alternative effect sizes and existing tools are outdated and inaccessible. In this paper, I introduce a free-to-use web-based calculator (https://dczhang.shinyapps.io/expectancyApp/) for generating alternative effect size displays from empirical data. This calculator requires no mathematical or programming expertise, and therefore, is ideal for academics and practitioners. I also present results from an empirical study that demonstrates the benefits of alternative effect size displays for enhancing lay people's perceived understandability of validity information and attitudes toward the use of standardized testing for college admissions.

“*The idea that expectancy tables, or other methods that go beyond simply reporting the correlation coefficient, may more clearly show the value of selection tests is not a new one, but it is an idea that must be regularly rediscovered as test critics continue to focus on variance accounted for*” – (Bridgeman et al., 2009)

Effect size statistics are universal in the social and behavioral sciences. In the academic literature, standardized effect size indices—such as the correlation coefficient or coefficient of determination—provide a metric for describing the strength of association between two variables (e.g., standardized test scores and academic performance) or the effect of an intervention (e.g., job training and job performance). Standardized indices serve as a common metric for comparing results across scientific studies. Understanding the magnitude of an effect allows decision makers to make informed decisions in domains such as employee selection, health intervention, and education policy.

Real world decisions, however, are often made by non-academics in context-rich environments. Standardized effect size indices preclude any contextual information and require statistical expertise to interpret them. These indices also tend to obscure the practical impact of a statistical effect. Lawshe and Bolda (1958), for instance, commented that the correlation coefficient does not clearly communicate the “predictive efficiency” of a variable. As a result, lay decision makers often find standardized statistical effect sizes hard to comprehend and ineffective for communicating the practical implications of research findings (Brogden, 1946; Rosenthal and Rubin, 1982b; Soyer and Hogarth, 2012; Highhouse et al., 2017). Given the limitations of standardized effect size statistics, there is considerable need to explore alternatives. Indeed, Mattern et al. (2009) commented that “…the ability to communicate validity research findings in a way that is meaningful to the general public should a be a top concern and priority for researchers” (p. 230).

To date, researchers have introduced several alternatives for translating traditional effect size statistics into more meaningful metrics (Lawshe and Bolda, 1958; Rosenthal, 1991; McGraw and Wong, 1995). Although these alternative metrics were developed to facilitate the interpretation of statistical effect size for non-experts, there are—unfortunately—no easily available tools for non-experts to produce these indices. Commonplace statistical programs, such as SPSS or *R*, do not readily generate alternative effect size statistics. Existing tools are outdated and inaccessible. Myors (1994), for instance, created a computer program for calculating theoretical expectancy charts but the original paper has never been cited. Similarly, Dunlap (1999) developed a program for calculating Common Language Effect Sizes (CLES) and has only been cited 28 times as of writing. Moreover, both programs were developed in FORTRAN, which is a severely outdated programing language. Some other existing tools require programming experience and do not provide a comprehensive list of common alternative effect size statistics (Table 1).

**Table 1:** Tools for calculating alternative effect sizes.

**Tool**	**Platform**	**Programming required**	**Effect size included**			**References**
			**Expectancy chart/table**	**CLES**	**BESD**	
Expectancy table calculator	FORTRAN	No	Yes	No	No	Myors, 1994
CLES calculator	FORTRAN	No	No	Yes	No	Dunlap, 1999
Expectancy chart calculator	*R*	Yes	Yes	No	No	Cucina et al., 2017
CLES calculator for Multiple Regression	*R*	Yes	No	Yes	No	Krasikova et al., 2018
**PRESENT APPLICATION**						
*Shiny-AESC*	*R* and *Shiny*	No	Yes	Yes	Yes	

*CLES, Common Language Effect Size; BESD, Binomial Effect Size Display*.

The lack of tools for computing these effect sizes may limit its proliferation in applied settings. As the opening quote illustrates, the reliance on traditional effect size indices such as correlations continues to obscure the practical impact of evidence-based selection tests: decades after the introduction of alternative effect size displays. The purpose of this paper is to introduce a web-based application that allows scholars and practitioners to easily generate and visualize a variety of alternative effect size metrics. The calculator does not require existing expertise in statistics or programming. In developing this tool, I hope to enhance the accessibility and visibility of alternative effect size displays in organizational and educational settings. Finally, I present an empirical experiment to illustrate the benefits alternative effect sizes have on the understandability of validity information and judgments toward evidence-based selection methods.

## Background

Traditional effect size indices, such as the correlation coefficient, are commonplace in the academic literature. Unfortunately, they are often difficult to understand and not easily translated into real-world outcomes. Moreover, the practical utility of correlations are often obscured: critics of using the SAT as a college admissions test asserted that “the SAT *only* adds 5.4 percent of variance explained by HSGPA [high school grade point average] alone” (Kidder and Rosner, 2002), even though the *same* evidence was used to support its utility in college admission decisions (e.g., Kuncel and Hezlett, 2007). Similarly, human resource professionals often judge academic literature as inaccessible and impractical (Terpstra and Rozell, 1998; Gelade, 2006), despite the abundance of validity data provided by academic researchers (Schmidt and Hunter, 1998; Kuncel et al., 2004; Huffcutt, 2011). Effect size information—when communicated effectively—should be easy to understand and elucidate the practical impacts of interventions or relations it aims to represent.

In response to the limitations of traditional effect size statistics, several alternative displays of effect size have been developed: the expectancy chart (Schrader, 1965), the Binomial Effect Size Display (Rosenthal and Rubin, 1982a) and the Common Language Effect Size (McGraw and Wong, 1992). Expectancy charts communicate the relationship between two variables (e.g., ACT score and GPA) by presenting the proportion of the sample with score above a cut-off criterion (e.g., GPA above 3.5) at a given score interval on the predictor (e.g., ACT score between 24 and 26). Similarly, the BESD uses a 2 × 2 matrix to present the linear relation between two variables as the probability of an outcome (e.g., GPA above 3.5) based on one's standing on a dichotomized predictor (e.g., ACT above vs. below 26). The theoretical values in the cells can be calculated with the equations: $\left(0.5+\frac{r}{2}\right)$ and $\left(0.5-\frac{r}{2}\right)$ where *r* is the bivariate sample correlation between the two variables of interest (Rosenthal and Rubin, 1982a). The CLES describes the difference between two groups (e.g., control vs. intervention group) with the probability that a random score from one group will differ from the control group (McGraw and Wong, 1992, also see Improvement Index, Clearinghouse, 2014). CLES can be calculated based on the mean and standard deviations of the groups. The effectiveness of the SAT training program, for example, can be described in CLES as “there is a 60% chance that a score from someone who took the ACT training will be better than someone without training.” Dunlap (1994) extended the original CLES to describe the relationship between two continuous variables where the comparison groups are operationalized as the subset of the sample with predictor scores above vs. below a cut-off. For example, the relationship between ACT scores and college GPA can be described as “there is a 60% chance that a student with an ACT score above the median will have a higher college GPA than a student with an ACT score below the median.”

## Empirical vs. theoretical effect sizes

Existing methods of computing alternative effect size metrics rely on computationally transforming an observed traditional effect size (e.g., sample correlation) into a theoretical alternative effect size (e.g., hypothetical expectancy chart). The resulting proportions and probabilities in the alternative effect size are not the actual values in the data, but rather, theoretical population values based on the observed sample correlation. The distinction between empirical and theoretical methods of computing alternative effect sizes is particularly salient in the development of expectancy charts (Lawshe and Bolda, 1958; Lawshe et al., 1958). Tiffin and Vincent (1960) found that hypothetical expectancy charts are appropriate when the sample size is adequately large. Existing calculators also use the hypothetical method for computing alternative effect sizes (e.g., Myors, 1994; Cucina et al., 2017).

Although hypothetical effect sizes may be more representative of population parameters, there are several theoretical and practical drawbacks for validity communication. First, when theoretical proportions are computed from a correlation coefficient, the results do not always reflect the data. Rosenthal (1991), for example, showed that the same correlation coefficient can produce markedly different proportions in the BESD and CLES depending on the cut-off values in the criterion and the sample sizes within criterion ranges. Therefore, it might inaccurate and possibly disingenuous to present stake holders with proportions of expected outcomes when those proportions are not, in fact, actual proportions in the data. Secondly, theoretical effect sizes require the audience to understand the difference between sample vs. population parameters: a distinction that may not resonate with a statistically naïve population. Baldridge et al. (2004) argued that “data should be described in a way that fits with how practitioner would describe the *situation* being addressed in the study.” Proportions presented “as is” (i.e., empirically derived from the sample) may alleviate an additional barrier to comprehension for non-experts. Third, hypothetical transformations of an effect size assume a linear relationship between two variables. This assumption is particularly relevant in expectancy charts, where a theoretical expectancy chart will necessarily depict a linear change in desired outcome as a function of the predictor. However, researchers have started to discover non-linear trends between predictors and criterion in applied settings [e.g., personality and job performance, (Carter et al., 2014)]. When non-linear trends are expected to exist in the data, theoretical transformations of traditional effect sizes are no longer appropriate. In contrast, empirically based displays may be more informative for observing possible curvilinear relationships. Based on these advantages, an empirical alternative effect size calculator was created. In the next section, I describe the development of a web application aimed at producing alternative effect size displays from empirical data.

## *Shiny-AESC* web app

The Alternative Effect Size Calculator (*Shiny-AESC*^1^ is created using *Shiny* (https://www.rstudio.com/products/shiny), which is a web application framework in the statistical programming language *R*. The popularity of *R* has risen considerably over the years (Robinson, 2017). *Shiny* allows developers to implement statistical procedures from *R* into user-friendly online applications. Users of *Shiny* apps do not need existing knowledge of *R*. *Shiny* is particularly useful for developing dynamic and interactive visualizations of data (Ellis and Merdian, 2015).

**Figure 1:**
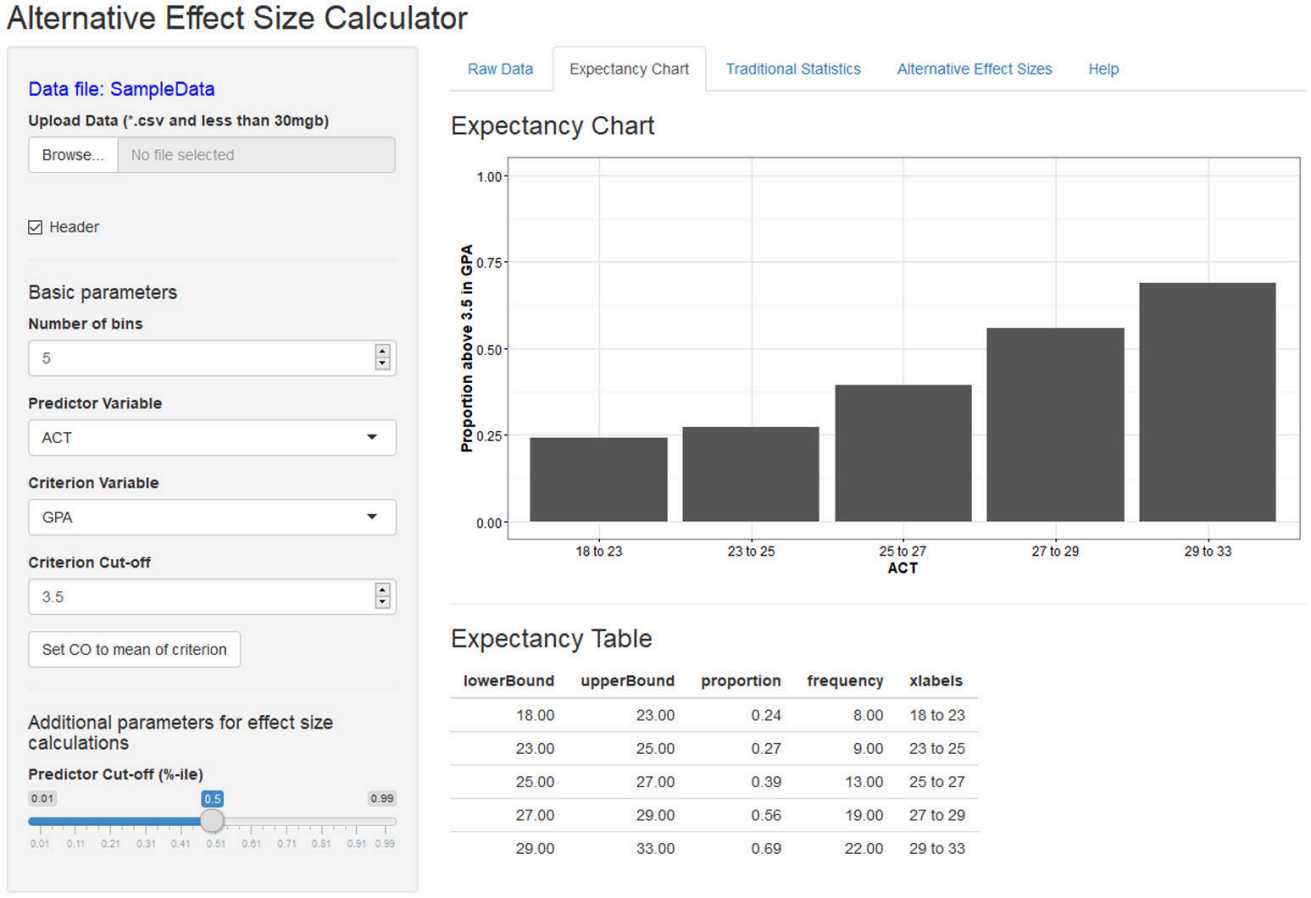
Screenshot of the *Shiny-AESC* main screen and expectancy chart output.

**Figure 2:**
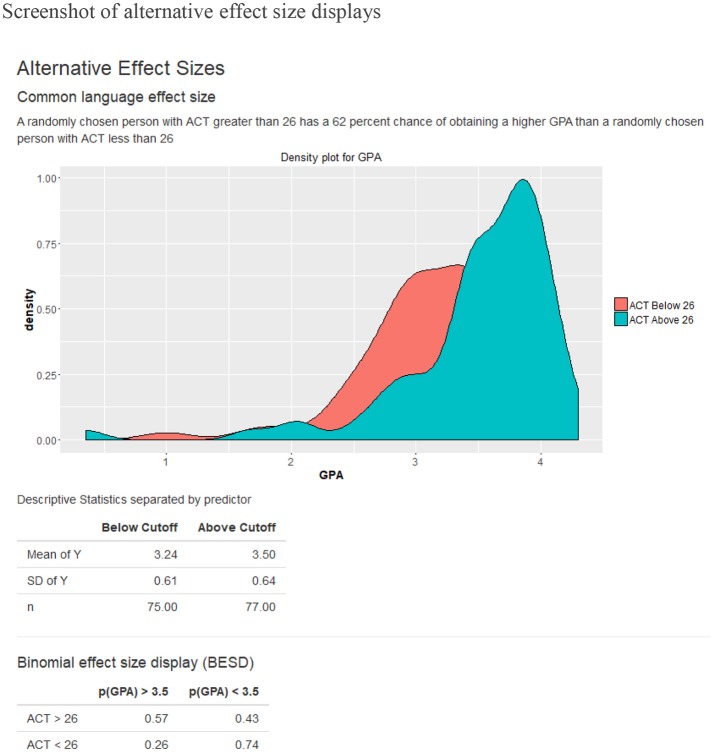
Screenshot of alternative effect size displays.

The *Shiny-AESC* is separated into the two sections: (1) the input panel, which allows users to upload their own data and manipulate various parameters for the effect size calculation; and (2) the output panel, which displays a variety of statistical information related to the variables. The output panel is separated into five tabs: expectancy chart, raw data, traditional statistics, alternative effect sizes, and help. Figures 1, 2 contains screenshots of the web app in the expectancy tab and the effect size tab. The presented web app is fully interactive, and the effect size outputs are dynamically generated based on the user input. In other words, as the user changes the input parameters, the resulting effect size displays are updated in real time.

Interactive data visualizations have several benefits for statistical comprehension. Interactivity refers to giving user control over the graphical elements in the visual display (e.g., words, pictures; Mayer and Chandler, 2001), which serves to enhance the engagement of the viewer (Perez and White, 1985; Rieber, 1990; Ancker et al., 2011). Moreover, interactive graphs often contain animations, which are ideal for communicating changes or trends over time or across categories (Morrison et al., 2000). Effectively implemented user-interactivity can reduce the viewer's cognitive load and lead to better learning outcomes (Mayer and Chandler, 2001). For instance, Boucheix and Guignard (2005) found that user-interactivity improved performance on the comprehension of a technical manual. Mayer and Chandler (2001) found that modest amount of interactivity improved students' learning of scientific concepts. The recent technological developments in web-based applications has allowed for more user-interactive platforms to manipulate and visualize data (Tay et al., 2016). Given the benefits of user-interactivity, some researchers have advocated the development of interactive data visualization tools to enhance statistical communication (Ellis and Merdian, 2015).

## Demonstration of *Shiny-AESC* with ACT data

The *Shiny-AESC* is used to generate displays of the validity of ACTs as a predictor of college GPA using archival data. The sample bivariate correlation between ACT and GPA in the dataset is 0.295. The expectancy chart was created with criterion cut-off set to a GPA of 3.5. I chose 3.5 because it is—for many universities in the United States—the cut-off value for being on the dean's list. It is notable that the average GPA in the sample was 3.35. Cut-off values for the predictor is also required for calculating the BESD and CLES. By default, the median value is used to ensure symmetry of sample sizes. To be sure, both predictor and criterion cut-offs are free to vary at the discretion of the user. Figure 1 depicts the association between ACT and GPA as an expectancy chart, and Figure 2 contains the BESD and CLES for the same relation.

## Empirical study: perceptions of validity comprehension and ACT usefulness

To illustrate the psychological benefits of alternative displays of effect sizes, I conducted an experiment that examined the effects of alternative vs. traditional effect size displays on the perceived understandability of validity evidence and people's attitudes toward the use of ACTs as a college admissions test. Standardized testing, including but not limited to ACTs, have received considerable criticism from academics and the public (Sternberg, 2006). Despite a substantial body of evidence in favor of the standardized tests as a valid predictor of college performance, scholars and college administrators often judge these tests as unfair and useless (Sherley, 2007). Many universities and colleges across the countries have started adopting a test-optional policy (Belasco et al., 2015). Proponents of the standardized tests have suggested that their perceived validity may be undermined by the choice of statistic (Mattern et al., 2009). In their report, Mattern et al. (2011) used primarily expectancy charts when presenting the validity of their admissions test; likewise, Kuncel and Hezlett (2007) used a BESD in response to criticisms of the magnitude of cognitive ability tests' predictive validity.

The benefits of alternative effect size statistics have also been shown in controlled experiments. Brooks et al. (2014), for instance, found that people perceived alternative effect sizes as easier to understand than traditional effect size indices (*r* and *r*^2^). Moreover, the authors found that the perceived utility of a training program was greater when its validity was presented with alternative effect sizes. In this experiment, I will present a lay audience with validity of ACTs presented as five different effect size metrics generated from the calculator (*r, r*^2^, BESD, CLES, and Expectancy Chart) and ask participants to report their understandability of the validity information and the usefulness of ACTs. The experiment is a conceptual replication and extension of findings from Brooks and colleagues. This study extends the previous study in two ways: (1) in additional to BESD and CLES, I will also examine the effect of the expectancy chart on validity communication; and (2) whereas Brooks and colleagues used theoretically derived effect sizes, this experiment will use empirically calculated effect size. Consistent with previous findings, I expect that:

*Hypothesis 1*. People will perceive alternative effect sizes (BESD, CLES, and Expectancy Chart) as easier to understand than traditional effect size statistics (*r* and *r*^2^).
*Hypothesis 2*. The ACT will be judged more favorably as a college admissions test when its validity is presented with alternative effect sizes displays than with traditional effect size statistics.

## Methods

### Sample

Adult participants (*n* = 225) from the United States were recruited using Amazon Mechanical Turk (MTurk). Past research has demonstrated that the MTurk population generalizes well to an adult population and is a valuable platform for conducting experimental research (Buhrmester et al., 2011; Highhouse and Zhang, 2015). Fifty-three participants were dropped for incorrectly responding to our quality check question (“ACT is a test of students' physical abilities”). The final sample had 172 participants (53% male, mean age = 36, 80% Caucasian). Each participant received 75 cents for completing the survey, which took approximately 5 min.

### Procedure

Participants first read a short description of the ACT as a standardized college entrance exam in the United States. Next, they were presented with information regarding the validity of the ACT. Each participant was randomly assigned to one of five conditions, each corresponding to a different type of validity display (correlation; coefficient of determination; CLES; BESD; and Expectancy Chart)^2^ Finally, participants reported—on 5-point Likert scales—the degree to which they understood the validity information using a four-item measure (e.g., “I understood the information about the relationship between ACT score and college GPA”) and the degree to which they judged the ACT as a useful tool for making college admission decisions using a three-item measure (e.g., “I would endorse the use of ACTs for admission purposes”). Both measures were adapted based on Brooks et al. (2014) and had Cronbach α values of 0.92 and 0.93 respectively. Appendix A contains all the items used in this study. For exploratory purposes, I also measured participants' numeracy using the four-item Berlin Numeracy Test (Cokely et al., 2012) and their self-reported ACT score.

## Results

Table 2 contains the means, standard deviations, and intercorrelation of the study's measures. Perceived comprehension and perceived utility of the ACT were moderately correlated. Self-reported ACT score was moderately correlated with scores on the Berlin Numeracy Test. Both scores were positively correlated with perceived comprehension of the statistical evidence, but not with the perceived utility of the ACT. It is notable that people who scored higher on the ACT did not necessarily exhibit better reaction toward the test (e.g., Wright et al., 2012).

**Table 2:** Means, standard deviations, and correlations of study variables.

**Variable**	***M***	***SD***	**1**	**2**	**3**	**4**	**5**
1. Validity comprehension	4.05	0.95					
2. ACT usefulness	3.64	0.95	0.31^*^				
3. Reported ACT score	3.93	1.46	0.27^*^	0.04			
4. Berlin numeracy	1.82	1.20	0.21^*^	0.08	0.36^*^		
5. Sex	1.47	–	−0.02	−0.03	0.08	−0.08	
6. Age	35.72	11.36	0.09	0.08	−0.01	0.08	0.09

* *Indicates p < 0.01*;

*M and SD are used to represent mean and standard deviation, respectively. Sex is coded as 1, Male; 2, Female*.

The purpose of the study was to examine the effects of traditional (*r* and *r*^2^) and alternative (CLES, BESD, and Expectancy Chart) validity displays on their perceived comprehension and subsequent judgments toward the ACT. Therefore, I collapsed the display conditions into traditional vs. alternative effect size displays for the ease of exposition. Table 3 contains the results of the Welch's *t*-tests^3^ for both dependent variables. There was a statistically significant difference in the perceived comprehension of the validity information. As expected, alternative effect sizes were perceived as easier to understand than traditional effect sizes. There was also a statistically significant effect of display type on the perceived usefulness of the ACT. People judged the ACT to be more useful for making college admission decisions when the validity information was presented as an alternative effect size display than a traditional effect size statistic. Parallel Bayesian independent *t*-tests were also conducted using JASP (JASP Team, 2018; Wagenmakers et al., 2018). Observed Bayes Factors for the alternative hypotheses (BF_10_) were greater than 100 for both dependent variables. In other words, the alternative hypotheses were 100 times more likely than the null hypotheses. Based on the results, Hypotheses 1 and 2 were fully supported.

**Table 3:** Independent samples *t*-test of dependent variables.

**Dependent variable**	***t*-statistic**	***df***	***p***	**Cohen's *d* [95% confidence interval]**
Perceived comprehension	5.35	111	<0.001	0.885 [0.56–1.19]
Perceived usefulness	3.78	123	<0.001	0.611 [0.29–0.91]

**Figure 3:**
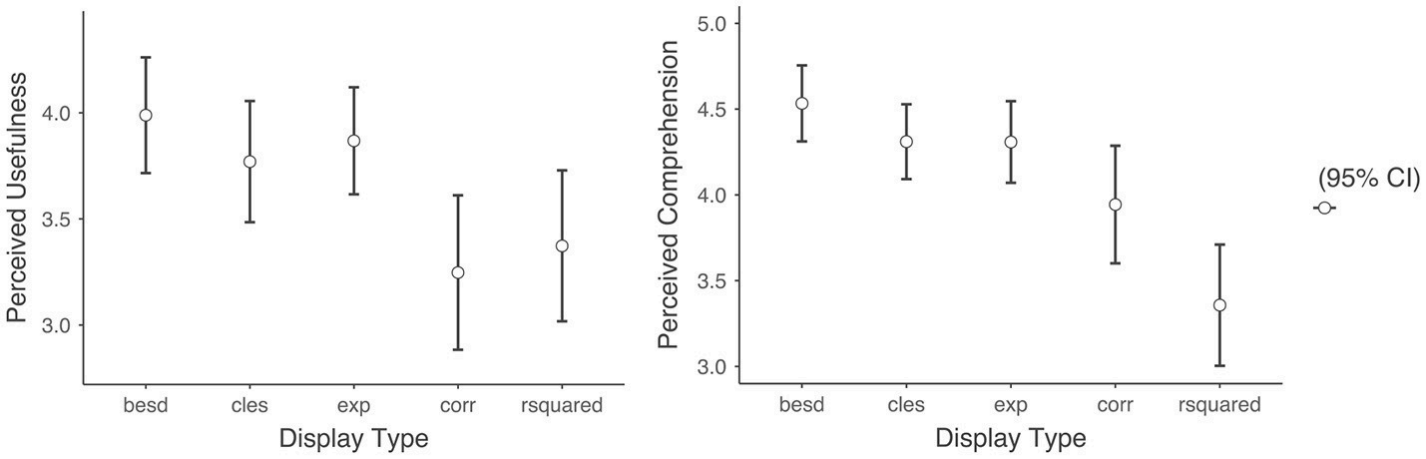
Means of perceived comprehension and perceived usefulness across display types. Besd, binomial effect size display; cles, common language effect display; exp, expectancy chart; corr, correlation coefficient; rsquared, coefficient of determination.

For exploratory purposes, I also examined the difference in the dependent variables between each of the display types (Figure 3). There were no noticeable differences in the perceived comprehension nor perceived usefulness of the ACT across the different alternative effect size displays. Although there was a noticeable difference in the perceived comprehension between the correlation and coefficient of determination, this difference was not statistically significant after correcting for multiple comparisons.

## Discussion

In the empirical experiment, people judged the alternative effect size indices as easier to understand than traditional effect sizes. The results are consistent with previous investigations of alternative effect sizes (Brooks et al., 2014). I also extended previous research by examining the expectancy chart, which is frequently used in organizational and education settings. I did not find any differences in the understandability between the expectancy chart and other alternative displays (CLES and BESD). Interestingly, there was a small—but not statistically significant—difference in the perceived comprehension between the correlation and coefficient of determination. One explanation is that the term “correlation” is used more frequently in non-academic settings, whereas “coefficient of determination” and “variance” are both technical concepts that are rarely mentioned outside the context of scientific research. Still, the differences in perceived comprehension did not translate to differences in attitude toward the ACT. Most importantly, participants of this study judged the ACT to be more useful when its validity was presented as one of the alternative effect size displays. The results suggest that the reluctance to use standardized tests in admission settings may be due—in part—to the way in which academics communicate validity information.

To facilitate the generation of alternative effect size statistics using empirical data, I have developed a working web-based interactive alternative effect size calculator (https://dczhang.shinyapps.io/expectancyApp/). The calculator will read in raw data and produce a series of traditional (e.g., Pearson's *r, r*^2^, Hedges' *g*) and alternative (e.g., BESD, CLES, Expectancy Chart) effect size displays. At the current stage, the calculator will only produce alternative effect sizes based on two continuous variables. Future developments of the calculator will allow for the calculation of the combined and incremental validity of multiple predictors (e.g., Bridgeman et al., 2004; Krasikova et al., 2018).

Although I have listed several benefits of empirically derived alternative effect sizes, it is important to note its limitations. Specifically, alternative effect sizes derived directly from the data are subject to sampling error and the observed patterns are subject to the influences of cut-off values and number of bins. The number of bins in the expectancy chart, for example, could either exacerbate or mask the observed relations, especially when empirical data is used. Indeed, there are numerous ways in which the disingenuous presentation of data can mislead the receivers of the information (Best, 2001; Parikh, 2014). Therefore, as with any decisions of displaying data, the user must carefully choose meaningful parameters. Future research should also examine differences in the interpretation of hypothetical vs. empirically derived alternative effect sizes, particularly with respect to the perceived believability and relevance of the results. Finally, the nature of the convenient sample may limit the empirical experiment. Future empirical research should examine the effect of validity displays of ACTs on a more invested population (e.g., policy makers, educators, parents).

This research has several practical implications. First, the *Shiny-AESC* provides practitioners and researchers with an easy-to-use tool for creating alternative effect size displays from empirical data. Practitioners are encouraged to use this tool when communicating validity evidence to relevant stakeholders and organizational decision-makers. Second, researchers are encouraged to include alternative effect size displays in their scientific publications. As shown in this paper and previous research, alternative effect sizes are easier to understand for a non-expert population and are more effective in communicating the practical benefit of the research findings. By presenting research evidence with more accessible metrics, we may finally begin to bridge the gap between academic research and practice in applied areas of psychology.

## Ethics statement

This study was carried out in accordance with the recommendations of the Institutional Review Board at Louisiana State University. The protocol was approved by the Institutional Review Board at Louisiana State University. All subjects gave written informed consent in accordance with the Declaration of Helsinki.

## Author contributions

The author confirms being the sole contributor of this work and approved it for publication.

### Conflict of interest statement

The author declares that the research was conducted in the absence of any commercial or financial relationships that could be construed as a potential conflict of interest.

## Footnotes

^1^Link to the app (https://dczhang.shinyapps.io/expectancyApp/).

^2^Stimulus material can be found on osf.io (https://bit.ly/2JVa3lO).

^3^Welch's *t*-test was used to accommodate unequal group sizes between the traditional (*n* = 73) and alternative effect size (*n* = 102) groups.

## Appendix A. Items of dependent variables

Perceived Comprehension

1. It was easy to understand the information about the effectiveness of the ACT
2. The relationship between ACT and college GPA was clearly communicated
3. I understood the information about the relationship between ACT score and college performance
4. I understood the value of the ACT as a tool for predicting college GPA

Perceived Usefulness

1. There is a strong association between ACT scores and college GPA
2. The ACT is an accurate tool for predicting college GPA
3. The ACT score is a strong predictor of college GP
